# Altered Resting-State Amygdala Functional Connectivity after 36 Hours of Total Sleep Deprivation

**DOI:** 10.1371/journal.pone.0112222

**Published:** 2014-11-05

**Authors:** Yongcong Shao, Yu Lei, Lubin Wang, Tianye Zhai, Xiao Jin, Wei Ni, Yue Yang, Shuwen Tan, Bo Wen, Enmao Ye, Zheng Yang

**Affiliations:** 1 Beijing Institute of Basic Medical Sciences, Beijing, PR China; 2 Cognitive and Mental Health Research Center, Beijing, PR China; Laureate Institute for Brain Research and The University of Oklahoma, United States of America

## Abstract

**Objectives:**

Recent neuroimaging studies have identified a potentially critical role of the amygdala in disrupted emotion neurocircuitry in individuals after total sleep deprivation (TSD). However, connectivity between the amygdala and cerebral cortex due to TSD remains to be elucidated. In this study, we used resting-state functional MRI (fMRI) to investigate the functional connectivity changes of the basolateral amygdala (BLA) and centromedial amygdala (CMA) in the brain after 36 h of TSD.

**Materials and Methods:**

Fourteen healthy adult men aged 25.9±2.3 years (range, 18–28 years) were enrolled in a within-subject crossover study. Using the BLA and CMA as separate seed regions, we examined resting-state functional connectivity with fMRI during rested wakefulness (RW) and after 36 h of TSD.

**Results:**

TSD resulted in a significant decrease in the functional connectivity between the BLA and several executive control regions (left dorsolateral prefrontal cortex [DLPFC], right dorsal anterior cingulate cortex [ACC], right inferior frontal gyrus [IFG]). Increased functional connectivity was found between the BLA and areas including the left posterior cingulate cortex/precuneus (PCC/PrCu) and right parahippocampal gyrus. With regard to CMA, increased functional connectivity was observed with the rostral anterior cingulate cortex (rACC) and right precentral gyrus.

**Conclusion:**

These findings demonstrate that disturbance in amygdala related circuits may contribute to TSD psychophysiology and suggest that functional connectivity studies of the amygdala during the resting state may be used to discern aberrant patterns of coupling within these circuits after TSD.

## Introduction

As extended work hours become a normal part of everyday life, many people are experiencing chronic sleep loss and sleep deprivation (SD). SD is associated with debilitating mental fatigue status characterized by hyperarousal and daytime sleepiness; it can lead to deficits in many cognitive capacities such as executive control, working memory, and psychomotor ability [1]–[3]. In addition, SD also causes negative emotion and affect, which may adversely impact brain function and result in human errors and accidents [4]–[8]. However, the exact brain mechanisms underlying SD’s influence on emotion remain to be elucidated.

The amygdala has a well-documented role in the emotional processing of salient information, especially aversive stimuli, and it has tight structural connections and reciprocal feedback loops with the medial prefrontal cortex (mPFC) and orbitofrontal cortex (OFC), as well as the dorsolateral prefrontal cortex (DLPFC) and anterior cingulate cortex (ACC) [9], [10]. Recent functional neuroimaging evidence highlights the amygdala as a structurally and functionally heterogeneous nucleus, with the basolateral amygdala (BLA) and centromedial amygdala (CMA) as two major nuclei that play important roles in emotional processing and generating behavioral responses. Neuroimaging studies have identified a potentially critical role of the amygdala in disrupted emotion neurocircuitry in individuals after total sleep deprivation (TSD) [5], [6], [11]. According to functional brain imaging studies that investigated the neural basis of emotional responses after acute SD, unpleasant emotional stimuli could increase amygdala activity after overnight TSD [7], [12]. By performing a psychophysiological interaction analysis of functional magnetic resonance imaging (fMRI) data, Banks and colleagues found that activity in specific areas of the frontal cortex (DLPFC, dorsal medial PFC [DMPFC], ACC, and OFC) covaried with amygdala activity in appraisal tasks [13]. These studies indicated that the amygdala might play a crucial role in emotion changes following TSD.

Because amygdala hyperactivity tends to coincide with PFC hypoactivity in healthy individuals, recent studies have begun to investigate functional connectivity between these regions [14]. Functional connectivity studies have provided additional and potentially more direct information about the regulatory relationships between specific PFC regions and the amygdala [13], [15]–[17]. Most SD neuroimaging studies have described abnormalities in regions involved in emotion regulation, including the mPFC, DLPFC, and ACC. Their results are consistent with the known role of the amygdala as a key region in threat detection, fear conditioning, and emotional salience, and the mPFC’s role as a modulatory region interconnected with limbic structures involved in emotion regulation [13], [18]. Moreover, recruitment of these frontal regions occurred when subjects engaged in active self-regulation and was associated with amygdala activity modulation [19], [20].

Taken together, previous fMRI studies suggest that TSD results in hyperactivation of the amygdala in response to emotion-related stimuli with corresponding hypoactivation of the mPFC, DLPFC, and rostral ACC [6], [11]. This pattern is generally understood to reflect a lack of regulatory control over emotion in individuals subjected to TSD. However, these relationships could be more clearly assessed by performing connectivity analyses at rest, which would eliminate the impacts of tasks that may elicit amygdala activity or provoke TSD influences.

Resting-state functional connectivity analysis is a powerful way to assess intrinsic connections between brain regions. This technique has revealed important functions such as processing speed and cognitive flexibility in health [21]. Recent studies have begun to investigate resting-state connectivity in individuals after TSD and have reported alterations in subcortical and default mode network (DMN) connectivity [22], [23]. Resting-state amygdala connectivity may have particular relevance for the study of emotion after TSD; clinical studies have revealed that it is altered in individuals with generalized anxiety disorder, social phobia, and major depressive disorder [24]–[26]. However, amygdala connectivity at rest in individuals after TSD is not fully understood. In this within-subject crossover study, BLA and CMA were used as seed regions to evaluate resting-state amygdala functional connectivity in male subjects during rested wakefulness (RW) and after 36 h of TSD. Giving that the PFC exerts regulatory control over the amygdala, we hypothesized that there would be reduced functional connectivity between PFC regions and the amygdala after TSD.

## Methods

The present investigation was part of a larger fMRI study that assessed the neural correlates of working memory after 36 h of TSD [27]. Only subjects with complete resting-state fMRI data were included in the current analyses. The study recruited 14 healthy, right-handed adult males (Mean ± SD age, 25.9±2.3 years) from Beijing Normal University as paid volunteers by advertisements. None of the subjects had previously participated in psycho-physiological experiments, and all had normal or corrected-to-normal vision. The exclusion criteria were as follows: diseases of the central and peripheral nervous systems, head trauma, cardiovascular diseases and/or hypertension, cataracts and/or glaucoma, pulmonary problems, or alcohol or drug abuse. None of the subjects showed evidence of clinical symptom levels as assessed by the Symptom Checklist-90 (SCL-90) with T-scores<60 on the General Symptom Index, and all the participants had normal intelligence scores (Raven test, intelligent quotient [IQ] >100) [28], [29]. All subjects were required to maintain a regular sleep schedule and refrain from alcohol, caffeine, and chocolate intake and napping for 1 week before the study and for its duration. After a complete description of the study was provided to the participants, written informed consent was obtained, and they established typical sleep patterns, defined as 8 h of sleep per night. This study was approved by the Research Ethics Committee of Beijing Institute of Basic Medical Sciences and the Fourth Military Medical University (Xi’an, China).

### Experiment paradigm

The experiment was carried out in the Basic Aerospace Institute with nursing staff present at all times. Each subject had a partner to help keep them awake through the night while under continuous behavior monitoring. Subjects were not allowed to leave the lab during the TSD period until they were escorted to the fMRI facility.

The participants were scanned twice, once during RW and once after 36 h of TSD. The two scanning sessions were conducted 3 weeks apart to minimize the possibility of residual effects of TSD affecting the cognition of volunteers who underwent the TSD scan before the RW scan. Both of the scan sessions were performed at the same time (8∶00 PM), and the scanning order was counterbalanced across subjects to reduce the potential influence of scan order.

### Resting-state paradigm

Participants underwent structural MRI and fMRI scanning that included resting-state procedures and working memory tasks. Participants were positioned in the scanner with their head comfortably restrained to reduce head movement. During the resting-state scans, subjects were instructed to keep their eyes closed, remain as motionless as possible, and not to think of anything in particular. A pulse oximeter was attached to the participant’s finger, allowing us to record their cardiac activity. In addition, participants wore a pressure belt around the abdomen to record their respiratory activity. The cardiac and respiratory signals were collected and synchronized to the fMRI data so that these physiologic variations could be removed during the regression analysis.

### Data acquisition

All MRI data were acquired at the General Hospital of the PLA of China. Structural and fMRI data were acquired on a GE 3.0 T Signa scanner with a birdcage RF imaging coil. The scanning sessions included: (i) localization, (ii) T1-fluid-attenuated inversion recovery (FLAIR) anatomy, (iii) a single resting-state session followed by several task-state fMRI sessions (Go/Nogo, working memory), and (iv) high-resolution spoiled gradient recalled echo (SPGR) anatomy. After participants were positioned in the scanner, 189 functional images were obtained at rest using an echo-planar imaging (EPI) sequence (echo time [TE] = 30 ms, repetition time [TR] = 2000 ms, field of view = 256×256 mm, slice thickness = 5 mm, slice gap = 1 mm, flip angle = 90°, matrix = 64×64, 20 oblique slices). Whole-brain anatomical images were acquired using a high-resolution SPGR sequence. To make sure that the subjects did not fall asleep during the scan, they were reminded to stay awake through the microphone before each run. After each scan, subjects were asked whether they were awake in the previous run, and all the subjects confirmed that they were.

### fMRI preprocessing procedures

FMRI data was completed using Analysis of Functional NeuroImages (AFNI) software (AFNI, http://afni.nimh.nih.gov/afni/) and FSL 5.0 (http://fsl.fmrib.ox.ac.uk/fsl/fslwiki/). For fMRI image preprocessing, the first 10 data points of resting-state datasets were discarded due to instability of the initial MRI signal while the subjects adapted to the circumstances. Cardiac and respiratory noise was regressed out by 3dretroicor (AFNI). Due to the incomplete physiological data of 1 subject, the final dataset included 13 subjects for further analysis. This was followed by despiking (compression of extreme time series outliers using a hyperbolic tangent function), volume registration, motion correction, and spatial smoothing (Gaussian kernel of full width at half maximum of 6 mm). A set of regressors, including the signal averaged over the white matter mask, cerebrospinal fluid mask, and six motion vectors and their first derivatives, were regressed out of the EPI time series. Then a band-pass filter was applied to maintain only low frequency fluctuations between 0.015 and 0.1 Hz.

### Functional connectivity analysis

The BLA and CMA are two major amygdala nuclei that contribute to emotional processing and the generation of behavioral responses [30]–[32]. Therefore, the BLA and CMA were used as seed regions to investigate the functional connectivity pattern of the amygdala after sleep deprivation. Regions of interest (ROIs) were determined using stereotaxic, probabilistic maps of cytoarchitectonic boundaries developed by Amunts [33] and implemented in FSL’s Juelich histological atlas, consistent with Roy’s study [30]. ROIs were created in the standard space, only including voxels with a probability of at least 50% of belonging to each subdivision (BLA, CMA). Then, the BLA and CMA were employed as seed regions in further separate functional connectivity analyses. Next, the mean time series of all voxels within the ROIs were extracted using 3dmaskave (AFNI).

The mean time course generated from the ROIs was correlated to the time courses of all brain voxels using Pearson cross-correlation. Next, Fisher’s z transform analysis was applied to the Pearson correlation coefficients to obtain an approximately normal distribution (z = 0.5ln[1+r]/[1–r]).

Separate group statistical analyses were conducted for amygdala connectivity maps from the right and left amygdala seeds. A one-sample *t*-test was employed to assess the whole-brain functional connectivity of the left and right amygdala (*p*<0.01, AlphaSim corrected). Then, a paired *t*-test was conducted to compare functional connectivity differences between the RW and TSD scans. Cluster size was determined using AlphaSim (AFNI) to correspond to a false-positive rate of *p*<0.05 corrected for multiple comparisons within the ROIs.

## Results

### Physiological Data

The respiration and heart rates of all subjects were monitored throughout fMRI scanning. The average values of individual respiration and heart rates before and after SD were compared using paired *t*-tests. No differences were found in heart or respiratory rate between the RW and TSD conditions (Heart rate: RW = 68.42±7.26, TSD = 72.00±6.61, *t* [1], [13] = −1.500, *p* = 0.161; Respiratory rate: RW = 19.01±2.42, TSD = 18.42±2.62, *t* [1], [13] = −1.084, *p* = 0.300).

### Functional connectivity of the amygdala

The one-sample *t*-test results showed that the BLA connected to extensive subcortical structures and a few cortical regions, whereas the CMA was primarily connected with cortical structures.

#### BLA

**Figure 1:**
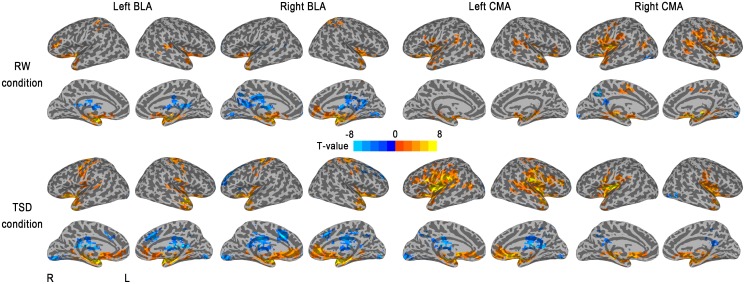
Whole-brain functional connectivity patterns of the BLA and CMA before and after 36-h TSD. Brain regions with positive correlations are displayed in warm colors, while negative correlations are displayed in cool colors.

The whole-brain functional connectivity patterns of the left and right BLA are shown in Fig. 1. The right BLA seed showed positive connectivity with a number of regions, including the bilateral parahippocampal gyri, bilateral superior temporal gyri (STG), and bilateral insula and negative correlations with the left posterior cingulate cortex (PCC), bilateral DLPFC, left dorsal ACC (dACC), and right medial frontal cortex.

The left BLA seed showed positive connectivity with the bilateral parahippocampal gyri, bilateral insula, and bilateral inferior frontal gyrus (IFG) and negative correlations with the right thalamus, right dACC, left declive, and left parahippocampal gyrus (Table 1 and Fig. 1).

**Table 1:** Activation results from single-group, whole-brain, voxel-wise analysis of the basolateral amygdale.

Brain regions	Cluster Size	Talairach coordinates			tscore
		x	y	z	
**Left BLA connectivity in TSD**					
Left Parahippocampal Gyrus	3175	+25.0	+7.0	–13.0	+19.05
Right Parahippocampal Gyrus		–25.0	+8.0	–13.0	+13.08
Right IFG		–32.0	–16.0	–22.0	+7.58
Left IFG		+36.0	–18.0	–18.0	+7.89
Right Insula		–40.0	+8.0	–6.0	+5.13
Left Insula		+38.0	+5.0	–6.0	+6.12
Right Thalamus	466	–11.0	+16.0	+16.0	–3.32
Left Precentral Gyrus	420	+40.0	+16.0	+53.0	+7.78
Left Declive	368	+7.0	+76.0	–10.0	–6.47
Right Postcentral Gyrus	345	–26.0	+31.0	+62.0	+7.48
Right dACC	189	–2.0	–17.0	+32.0	–5.58
**Left BLA connectivity in RW**					
Left Parahippocampal Gyrus	2287	+31.0	+4.0	–10.0	+13.89
Right Parahippocampal Gyrus		–28.0	+5.0	–14.0	+9.16
Right IFG		–31.0	–20.0	–22.0	+5.52
Left IFG		+37.0	–17.0	–18.0	+4.42
Right Lentiform Nucleus		–25.0	+1.0	–2.0	+4.75
Left Lentiform Nucleus		+31.0	+8.0	–2.0	+5.95
Left Parahippocampal Gyrus	376	+28.0	+49.0	+11.0	–10.20
Left IPL	91	+37.0	+43.0	+56.0	+5.97
Left dlPFC	88	+43.0	–32.0	+14.0	+5.72
Right MTG	71	–65.0	+40.0	+5.0	+5.70
**Right BLA connectivity in TSD**					
Right Parahippocampal Gyrus	3212	–29.0	+4.0	–13.0	+17.95
Left Parahippocampal Gyrus		+28.0	+1.0	–9.0	+13.69
Right STG		–32.0	–10.0	–21.0	+10.84
Left STG		+40.0	–6.0	–17.0	+7.11
Right ACC		–5.0	–32.0	+3.0	+5.68
Right Insula		–38.0	+5.0	–5.0	+7.20
Left Insula		+38.0	+4.0	–5.0	+4.67
Left dACC	798	+1.0	–20.0	+35.0	–9.27
Left dlPFC	514	+28.0	–29.0	+32.0	–7.09
Right Precentral Gyrus	383	–32.0	+22.0	+56.0	+6.68
Left Precentral Gyrus	234	+40.0	+19.0	+56.0	+8.31
Right Declive	203	–5.0	+79.0	–10.0	–6.96
Right dlPFC	172	–32.0	–44.0	+29.0	–6.45
Right Declive	59	–32.0	+67.0	–13.0	–5.61
**Right BLA connectivity in RW**					
Right Parahippocampal Gyrus	2443	–29.0	+4.0	–13.0	+23.81
Left Parahippocampal gyrus		+19.0	+5.0	–13.0	+9.36
Right STG		–35.0	–7.0	–29.0	+5.84
Left STG		+37.0	–20.0	–21.0	+5.85
Right Insula		–42.0	–9.0	–5.0	+4.15
Left PCC	766	+4.0	+46.0	+14.0	–5.94
Right Medial Frontal Gyrus	324	–17.0	–65.0	–4.0	–8.04
Right SPL	248	–32.0	+55.0	+53.0	+7.42
Right ACC	82	–8.0	–38.0	+2.0	+5.01
Left MTG	69	+37.0	+49.0	+11.0	–5.23

#### CMA

The whole-brain functional connectivity patterns of the left and right CMA are shown in Fig. 1. The right CMA seed showed positive connectivity with a number of regions, including the bilateral lentiform nucleus, bilateral insula, bilateral IFG, and bilateral STG and negative correlations with the left PCC, bilateral middle occipital gyrus, left medial frontal gyrus, and right lingual gyrus.

The left CMA seed showed positive connectivity with the bilateral lentiform nucleus, bilateral IFG, and bilateral insula and negative correlations with the right parahippocampal gyrus and right lingual gyrus (Table 2 and Fig. 1).

**Table 2:** Activation results from single-group, whole-brain, voxel-wise analysis of centromedial amygdale.

Brain regions	Cluster Size	Talairach coordinates			tscore
		x	y	z	
**Left CMA connectivity in TSD**					
Left Lentiform Nucleus	5854	+19.0	+7.0	–4.0	+20.12
Right Lentiform Nucleus		–28.0	+5.0	–4.0	+11.38
Right IFG		–29.0	–28.0	–4.0	+7.67
Left IFG		+35.0	–21.0	–12.0	+5.59
Right ACC		–8.0	–39.0	–4.0	+5.17
Right Insula		–37.0	+9.0	+8.0	+6.36
Left Insula		+38.0	+12.0	+8.0	+6.98
Left IPL		+54.0	+34.0	+24.0	+6.25
Right IPL		–58.0	+23.0	+24.0	+4.08
Right Parahippocampal Gyrus	524	–32.0	+49.0	+5.0	–6.93
Right Lingual Gyrus	290	–8.0	+70.0	–4.0	–7.83
Left MFG	64	+19.0	+16.0	+59.0	+5.65
**Left CMA connectivity in RW**					
Left Lentiform Nucleus	2149	+22.0	+10.0	–7.0	+55.19
Right Lentiform Nucleus		–23.0	+5.0	–3.0	+9.86
Right IFG		–30.0	–20.0	–11.0	+4.66
Left IFG		+32.0	–22.0	–7.0	+6.30
Right STG		–48.0	+7.0	–7.0	+7.23
Left MTG		+53.0	+9.0	–7.0	+5.95
Right Insula		–43.0	+8.0	–3.0	+3.79
Left Insula		+37.0	+9.0	+9.0	+4.21
Right STG	193	–50.0	+40.0	+14.0	+5.81
Left STG	133	+55.0	+55.0	+14.0	+5.32
**Right CMA connectivity in TSD**					
Right Lentiform Nucleus	3575	–26.0	+4.0	–4.0	+28.64
Left Lentiform Nucleus		+23.0	+5.0	–4.0	+12.56
Right Insula		–35.0	+10.0	+12.0	+4.40
Left Insula		+34.0	+5.0	+12.0	+7.73
Right IFG		–28.0	–15.0	–12.0	+4.83
Left IFG		+41.0	–16.0	–12.0	+6.39
Right Thalamus		–6.0	+15.0	+4.0	+5.43
Left PCC	131	+13.0	+43.0	+29.0	–6.99
Right Middle Occipital Gyrus	61	–47.0	+67.0	–7.0	–5.62
Left Medial Frontal Gyrus	58	+10.0	–65.0	+11.0	–4.42
**Right CMA connectivity in RW**					
Right Claustrum	4365	–32.0	+7.0	–7.0	+26.10
Right STG		–44.0	+3.0	–11.0	+5.42
Left STG		+48.0	–13.0	–11.0	+6.86
Right Insula		–41.0	+7.0	+1.0	+4.69
Left Insula		+34.0	+10.0	+13.0	+7.32
Right IPL		–61.0	+26.0	+25.0	+4.20
Right IFG		–53.0	–6.0	+29.0	+5.37
Left Medial Frontal Gyrus	103	+13.0	–62.0	+2.0	–6.66
Left dACC	103	+1.0	+1.0	+41.0	+5.02
Left MTG	98	+46.0	+58.0	+11.0	+5.11
Left PCC	94	+13.0	+49.0	+35.0	–15.45
Right Lingual Gyrus	63	–11.0	+85.0	–7.0	–6.87
Left Middle Occipital Gyrus	59	+46.0	+70.0	–7.0	–5.58

### Altered amygdala connectivity after TSD

#### BLA

**Figure 2:**
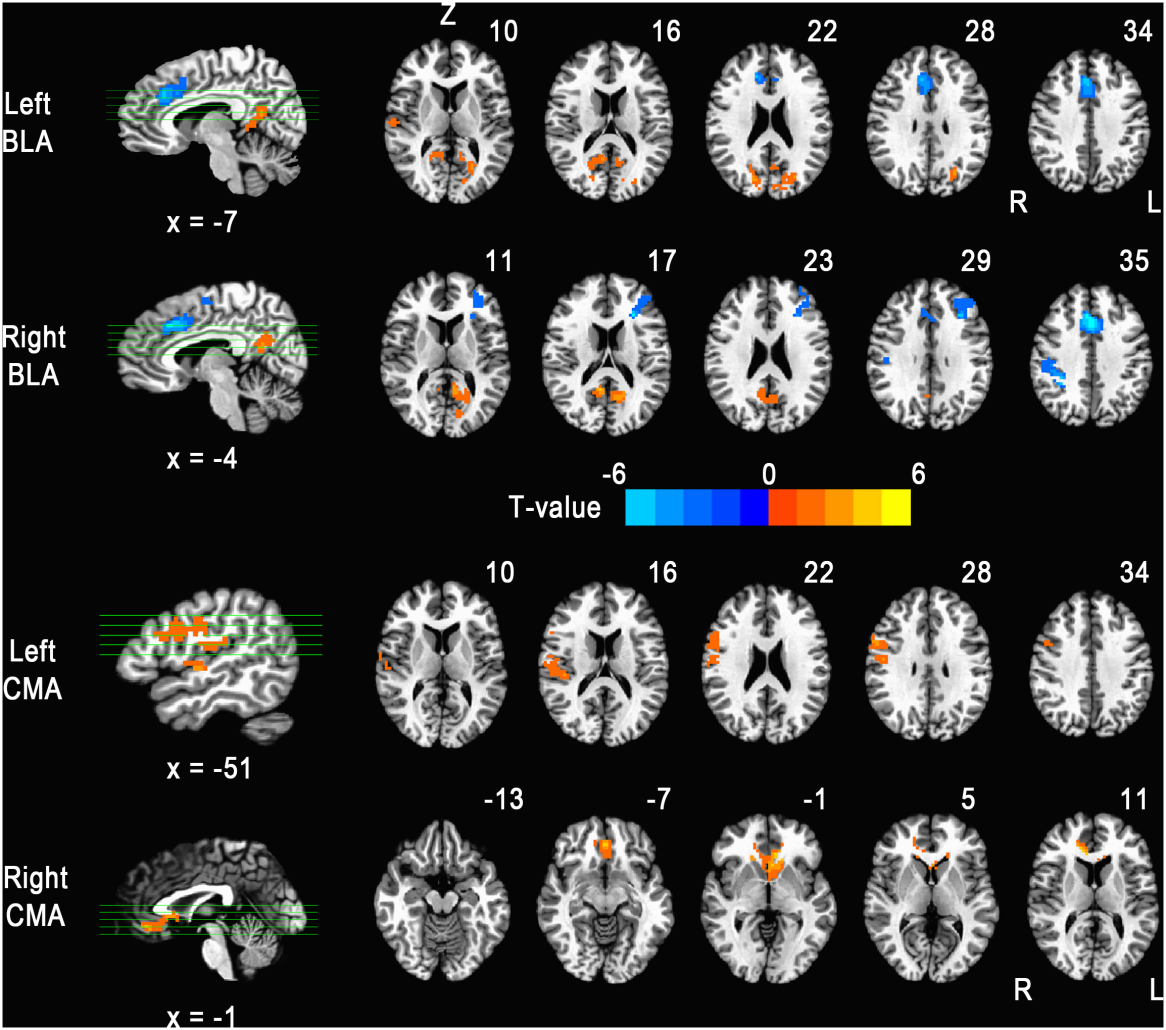
Brain areas that exhibited altered functional connectivity after 36 h TSD. The left sides of the images in transverse views represent the right hemisphere.

Compared with RW, participants after TSD showed reduced right BLA functional connectivity with the left DLPFC (Talairach coordinates x = 30.9, y = −36.5, z = 19.3; k = 208; t score = −2.32), right dACC (x = −0.9, y = −13.3, z = 38.6; k = 228; t score = −4.76), and right IFG (x = −37.6, y = 35.1, z = 41.1; k = 205; t score = −3.52) and reduced left BLA functional connectivity with the right dACC (x = −3.9, y = −20.1, z = 34.7; k = 238; t score = −3.50). After TSD, increased right BLA functional connectivity was observed with the left PCC/precuneus (PrCu) (x = 10.1, y = 53.8, z = 9.3; k = 320; t score = 2.45). Increased left BLA functional connectivity was found with the left PCC/PrCu (x = 19.3, y = 57.4, z = 10.4; k = 252; t score = 2.20) and right parahippocampal gyrus (x = −27.4, y = 33.9, z = 2.3; k = 355; t score = 2.83) (Table 3 and Fig. 2).

**Table 3:** Anatomical localization, cluster size, Talairach coordinates, and maximum T-values of TSD-induced functional connectivity changes.

Brain regions	Size	Talairach coordinates			T score
		x	y	z	
Seed: Left BLA (after TSDvs. before TSD)					
Right Parahippocampal Gyrus	355	–27.4	+33.9	+2.3	+2.83
Left PCC	252	+19.3	+57.4	+10.4	+2.20
Right dACC	238	–3.9	–20.1	+34.7	–3.50
Seed: Right BLA (after TSDvs. before TSD)					
Left PCC	320	+10.1	+53.8	+9.3	+2.45
Right dACC	228	–0.9	–13.3	+38.6	–4.76
Left dlPFC	208	+30.9	–36.5	+19.3	–2.32
Right IFG	205	–37.6	+35.1	+41.1	–3.52
Seed: Left CMA (after TSD vs. before TSD)					
Right Precentral Gyrus	222	–50.5	+6.2	+22.3	+2.68
Seed: Right CMA (after TSD vs. before TSD)					
Right rACC	246	–0.9	–25.9	+1.2	+2.19

#### CMA

Compared with rested wakefulness, participants after TSD showed increased right CMA functional connectivity with the right rostral ACC (x = −0.9, y = −25.9, z = 1.2; k = 246; t score = 2.19). After TSD, increased left CMA functional connectivity was found with the right precentral gyrus (x = −50.5, y = 6.2, z = 22.3; k = 222; t score = 2.68) (Table 3 and Fig. 2).

## Discussion

In this study, we investigated resting-state functional connectivity patterns of the BLA and CMA in whole-brain analyses and compared RW and TSD states. To our knowledge, this is the first examination of resting-state amygdala connectivity in healthy volunteers after TSD. Our observations of significantly reduced seed-based functional connectivity of the left DLPFC, right dACC, and right IFG with the BLA after TSD are particularly interesting as they bring prefrontal regions to the forefront of the neuronal mechanism of emotional disruption after TSD. In addition, participants also showed increased functional connectivity between the BLA and both the right parahippocampal gyrus and left PCC after TSD. Likewise, increased CMA functional connectivity was found in the right rACC and right precentral gyrus compared with the RW scan. The aberrant resting-state amygdala functional connectivity observed in this study supports the hypothesis of emotional dysfunction after TSD.

Prior neuroimaging studies of emotion regulation have posited that conscious down-regulation of emotion appears to have a top-down inhibitory effect on prefrontal brain regions, including the DLPFC [13], [34]. A study by Duerden et al. indicated that the DLPFC plays an important role in inhibition during cognitive-emotion inference tasks, suggesting that the DLPFC is involved in self-regulation and modulation of amygdala activity [35]. Our results documented a deficit in functional connectivity within emotional frontal-limbic networks after TSD, which is consistent with previous work [9]. Because the DLPFC appears to exhibit dysfunction during cognitive-emotional tasks in patients with depression, anxiety, or impulsive aggression, findings of reduced BLA-DLPFC functional connectivity support weakened emotion-regulation after TSD [24]–[26].

The extent of amygdala engagement can be influenced by the mPFC, a brain area proposed to exert inhibitory, top-down control of amygdala function, resulting in contextually appropriate emotional response [16]. Several studies found significantly reduced prefrontal-amygdala functional connectivity and emotional functioning after sleep loss [9], [36]. A study conducted by Yoo and his colleagues that included task performance suggested that a decline in functional connectivity between the amygdala and mPFC might reflect decreased inhibition of the frontal lobe after TSD [6]. Other lines of evidence support an executive control role of the DLPFC that suppresses amygdala activity [37]. According to the emotion regulation model described by Hermann, because there is no direct link between the DLPFC and amygdala, the DLPFC may integrate information from the mPFC and amygdala, regulates emotional expression [38].

In addition to the interesting finding of decreased functional connectivity between the BLA and left DLPFC, decreased BLA functional connectivities were also found with the right dACC and right IFG. The finding of reduced functional connectivity between the BLA and dACC is consistent with results of previous task-based functional connectivity studies [9]. Motomura et al. found that continuous and accumulating sleep debt could down-regulate functional suppression of the amygdala by the ACC [9]. In clinical studies, resting-state amygdala connectivities suggest that there is reduced functional connectivity between the PFC regions and amygdala in low-anxiety individuals and, interestingly, a lack of coupling in high-anxiety individuals [15]. Similarly, Yoo and colleagues reported that the PFC inhibited the amygdala during neutral emotion conditions, and this relationship was diminished in individuals with posttraumatic stress disorder [39], [40]. Considering the reduced functional connectivity between the PFC and amygdala supported by the above studies, we suggest that TSD may reduce the functional communication of brain emotion network. This is indeed consistent with a theory of reduced top-down regulation of the amygdala by emotional regulatory circuits [10]. As the relationship between emotional and cognitive deficits was not investigated in this study, poor cognition after TSD may also contribute to the dysregulation of emotion.

Additionally, increased functional connectivity was found between the BLA and areas including the left PCC/PrCu and right parahippocampal gyrus. With regard to the CMA, increased functional connectivity was found in the right rACC and right precentral gyrus. These regions are core areas of the default mode network (DMN), which is more active at rest than during goal-oriented tasks [41]. A recent study also reported increased functional connectivity between the amygdala and DMN regions in healthy individuals after psychosocial stress exposure [42]. As a key structure for emotion processing and regulation in the brain, the amygdala is functionally connected not only to the ACC and prefrontal cortices, but also to other brain regions including the parahippocampal gyrus, insula, MTG, and PCC/PrCu [30], [43]–[45]. The parahippocampal contribution to the DMN has been attributed to its involvement in episodic memory [46]. A number of studies have consistently suggested the important role of PCC/PrCu activity in emotion-related processes including emotion evaluation [47], happy and sad word processing [48], and social behavior [49]. A recent connectivity modeling analysis found a strong functional link between the PCC and amygdala in healthy individuals [30]. In this study, the TSD state was associated with a significantly increased correlation between the amygdala and both the PCC/PrCu and rACC compared to the RW state. Our findings suggest disruptions in these connections may point to DMN disturbances in subjects after TSD, a hypothesis that merits further research.

## Limitations

Our study has several limitations. Firstly, we only assessed male volunteers, so we cannot make generalizations to females. Only male volunteers were recruited due to the experimental conditions (paired subjects) and the long time course of the study. This will limit the clinical utility of the findings. In the future, it would be interesting to investigate sex differences in functional connectivity changes following TSD. Secondly, we interpreted our findings under the assumption that participants under both the RW and TSD states responded similarly to the scanning environment. However, it is possible that the participants experienced higher anxiety levels during the scan after TSD, and this could potentially contribute to the different connectivity patterns that we observed. Thirdly, verbal reporting of subjects’ in-scanner sleeping behavior is not an adequate measure. It is possible that some of the sleep-deprived participants may have actually fallen asleep during the resting state scan. In future experiments, we will consider the use of real-time EEG to monitor subjects’ in the scanner.

## Conclusion

These findings demonstrate that disturbance in amygdala related circuits may contribute to TSD psychophysiology and suggest that functional connectivity studies of the amygdala during the resting state may be used to discern aberrant patterns of coupling within these circuits after TSD.
